# MMP-9 as a clinical marker for endometriosis: a meta-analysis and bioinformatics analysis

**DOI:** 10.3389/fendo.2024.1475531

**Published:** 2024-10-31

**Authors:** Qiumei Huang, Yanlun Song, Xiaocan Lei, Hua Huang, Weihua Nong

**Affiliations:** ^1^ Department of Obstetrics and Gynecology, Affiliated Hospital of Youjiang Medical University for Nationalities, Key Laboratory of Research on Clinical Molecular Diagnosis for High Incidence Diseases in Western Guangxi, Baise, Guangxi, China; ^2^ Clinical Anatomy and Reproductive Medicine Application Institute, Hengyang Medical School, University of South China, Hengyang, Hunan, China; ^3^ Reproductive Hospital of Guangxi Zhuang Autonomous Region, Nanning, Guangxi, China

**Keywords:** matrix metalloproteinase 9, endometriosis, systematic review, meta-analysis, bioinformatics analysis

## Abstract

**Aim:**

This study systematically evaluated the potential efficacy of serum matrix metalloproteinase-9 (MMP-9) concentration as a diagnostic marker for endometriosis through meta-analysis. Early and accurate diagnosis of endometriosis, a common gynecological disease, is crucial for improving patient prognosis. Hence, this study aimed to comprehensively analyze the data from multiple studies to assess the diagnostic value of serum MMP-9 concentration for endometriosis.

**Methods:**

Articles investigating the association between MMP-9 and endometriosis, published from the inception of the databases until February 2024, were systematically retrieved from multiple databases, including PubMed, Embase, Cochrane, Web of Science, Scopus, and CNKI. Download and analyze the GSE7305, GSE23339, and GSE51981 datasets. Statistical analyses of all eligible studies were conducted using RevMan 5.4, Stata 11.0, and R software version 4.3.3.

**Results:**

Fifteen studies fully met the inclusion criteria for the meta-analysis. The concentration of MMP-9 in the blood of patients with endometriosis was significantly higher compared to that of the control group (*p* < 0.0001). Subgroup analysis based on different stages of endometriosis revealed a trend towards significantly higher serum MMP-9 concentrations in patients, whether in stages I-II or III-IV. Bioinformatics analysis revealed differences in the expression of MMP-9 in endometrial tissue between EMT patients and healthy controls in the GSE7305 and GSE23339 datasets. Additionally, in the GSE51981 dataset, we found significant differences between the normal group and both mild and severe cases of endometriosis.

**Conclusion:**

Both the current meta-analysis and bioinformatics analysis indicate differences in MMP-9 concentration levels between endometriosis patients and healthy individuals, with potentially elevated MMP-9 concentrations in serum samples from patients with endometriosis.

**Systematic review registration:**

https://www.crd.york.ac.uk/prospero, identifier CRD42024525864.

## Introduction

Endometriosis is a prevalent disorder of the female reproductive system marked by the presence of ectopic endometrial tissue in the pelvic cavity and peritoneum (1, 2). Patients commonly experience cyclical abdominal pain, infertility, and additional symptoms leading to a marked decline in quality of life (3–5). Although the exact cause of endometriosis is unknown, it is thought to be related to a number of factors including genetics, immunity and the environment (6).

Currently, the diagnosis of endometriosis relies on clinical symptoms, physical examination and imaging (3, 7–10). Nonetheless, these methods are limited by factors like invasiveness, challenges in detecting mild cases, and accurately evaluating lesion extent. Consequently, the quest for dependable biomarkers has emerged as a pivotal strategy to enhance diagnostic accuracy and facilitate early screening for endometriosis.

Matrix metalloproteinase-9 is an enzyme implicated in inflammatory responses and cell migration, potentially exerting a significant influence on endometriosis pathogenesis (11). Elevated serum MMP-9 levels correlate with the pathogenesis and clinical presentation of endometriosis (12), suggesting its potential utility as a serological diagnostic marker.

Nevertheless, findings from studies on the serological concentrations of MMP-9 in endometriosis and its diagnostic significance have been conflicting. While certain studies have reported a marked increase in serum MMP-9 levels among patients with endometriosis, others have found no significant differences. Hence, conducting a meta-analysis to comprehensively evaluate existing studies is crucial for clarifying the reliability and accuracy of MMP-9 as a serological diagnostic marker for endometriosis. The aim of this study was to systematically assess the serum expression of MMP-9 in the existing literature on endometriosis patients and to investigate its association with disease severity, clinical symptoms, and other influencing factors. This meta-analysis can provide more reliable theoretical support for the early diagnosis and treatment of endometriosis, and provide more beneficial treatment guidance for clinical practice.

## Materials and methods

The meta-analysis conducted in this study was registered with the International Prospective Registry of Systematic Reviews (PROSPERO, https://www.crd.york.ac.uk/prospero/display_record.php?ID=CRD42024525864).

The researchers systematically searched multiple databases (e.g., EMBASE, PubMed, Web of Science, Cochrane Library, Scopus, and CNKI) to identify relevant published articles that examined the relationship between matrix metalloproteinases and endometriosis during the period from the beginning of the database’s establishment to February 2024. The following search terms were used in this meta-analysis: “endometriosis” [MeSH], “endometriosis”, “endometrioma” and “endometrioma” and “matrix metalloproteinases” [MeSH], “metalloproteinases, matrix”, “MMPs”, “matrix metalloproteinases”, and “metalloproteinases, matrix”, and the detailed research strategy provided in **Supplementary Data Sheet 1**.

### Study selection and exclusion criteria

Inclusion criteria included (1): case-control studies, (2) laparoscopic diagnosis of endometriosis in patients classified according to the ASRM classification for Endometriosis; (3) inclusion of complete data or data that could be extrapolated from available results; (4) availability of full-text articles in English and Chinese; and (5) preference for reports with larger sample sizes or more detailed information in cases of multiple publications.

Exclusion criteria included: (1) failure to obtain adequate data from the original research article and inability to communicate with authors for detailed information; (2) letters, review articles, meta-analyses, conference papers, and animal studies; (3) articles with redundant data already incorporated in this study; (4) Non-ELISA detection methods were excluded.

### Literature screening, data extraction and quality evaluation

Following the search method outlined above, two researchers initially screened eligible articles based on their titles and abstracts. Subsequently, the selected articles underwent a thorough review of the full text. Any discrepancies between the two researchers were resolved by consulting a third researcher, and a final unanimous decision was reached through consensus.

### Data extraction and management

A table was constructed to extract the following information from eligible articles: (1) basic details, such as the first author and publication date; and (2) baseline characteristics of the study participants, including country, age, sample size, study type, measurement method, distribution of cases across different endometriosis stages (e.g., stages I-II, III-IV), and MMP-9 concentration.

### Quality assessment

The quality of the literature was assessed by two researchers utilizing the Newcastle-Ottawa Scale (NOS) (13). Disagreements in the quality assessment process were resolved through consultation with two additional reviewers or arbitration by an expert in the field. Each study was assigned a maximum score of 9, determined by criteria including study population selection (4 out of 4 items), comparability (1 out of 2 criteria), and exposure or outcome (3 out of 3 criteria). Scores fell into three ranges: 0 to 3, 4 to 6, and 7 to 9, denoting low, moderate, and high quality, respectively.

### Bioinformatics analysis

#### Data download and analysis of variances

Three microarray datasets (GSE7305, GSE23339, and GSE51981) related to endometriosis were retrieved from a public database on the GEO website (www.ncbi.nlm.nih.gov/geo) using the GEOquery R package (version 2.60). The GSE7305 dataset (GPL570 platform) contained endometrial tissue samples from 10 healthy individuals and 10 endometriosis samples for further analysis. The GSE23339 dataset (GPL6120 platform) includes 9 healthy tissue samples and 10 endometriosis tissues; The GSE51981 dataset (GPL570 platform) contained 71 matched healthy samples, 27 mild and 48 endometrial tissue from severe endometriosis samples, and validated analyses of the GSE7305 results were performed using GSE23339 and GSE51981. Convert the data into gene names using Perl software (https://www.perl.org/). Differential analysis of studied genes was performed using the ‘limma’ software package (version 3.52.1), and the results were visualized using the ‘ggpubr’ software package (version 0.6.0) and R software version 4.3.3.

### Gene set variation analysis and gene set enrichment analysis

The GSVA package (version 2.11) was used to import the relevant c2.cp.kegg.v7.4. and c5.go.bp.v7.5.1 files obtained from the Molecular Signatures Database (MSigDB) website database for further GSVA analysis. The clusterProfiler package and the enrichplot package were used for GSEA analysis. Meanwhile, the limma R package (version 3.52.1) was used to compare GSVA scores between different clusters and for GSEA analyses of the study genes.

### Statistical analysis

Data analyses were conducted using Stata 14.0 (Stata Corporation, College Station, TX, USA) and RevMan 5.4 (ReviewManager) software. Continuous data were estimated using weighted mean difference (MD), confidence interval (CI) was 95%, and *p* < 0.05 was considered statistically significant. To eliminate the impact of different units, standardized mean difference (SMD) was used for assessment. When *I^2^* < 50% or *p* > 0.1, fixed-effect model was used, indicating low heterogeneity of included studies. Conversely, if there is significant heterogeneity between the included studies, a random effects model is used. Sensitivity and meta-regression analyses were used to identify sources of heterogeneity and subgroup analyses were performed for control. Publication bias was evaluated by funnel plot and Egger and Begg tests were conducted by Stata 14.0 software. *p* > 0.05 indicated no significant publication bias. When the p-values of Egger test and Begg test conflict, the Egger test results are more convincing. Visualization and various statistical analyses utilized GraphPad Prism 9, RevMan 5.4, Stata 14, and R software version 4.3.3.

### Literature search and inclusion

**Figure 1:**
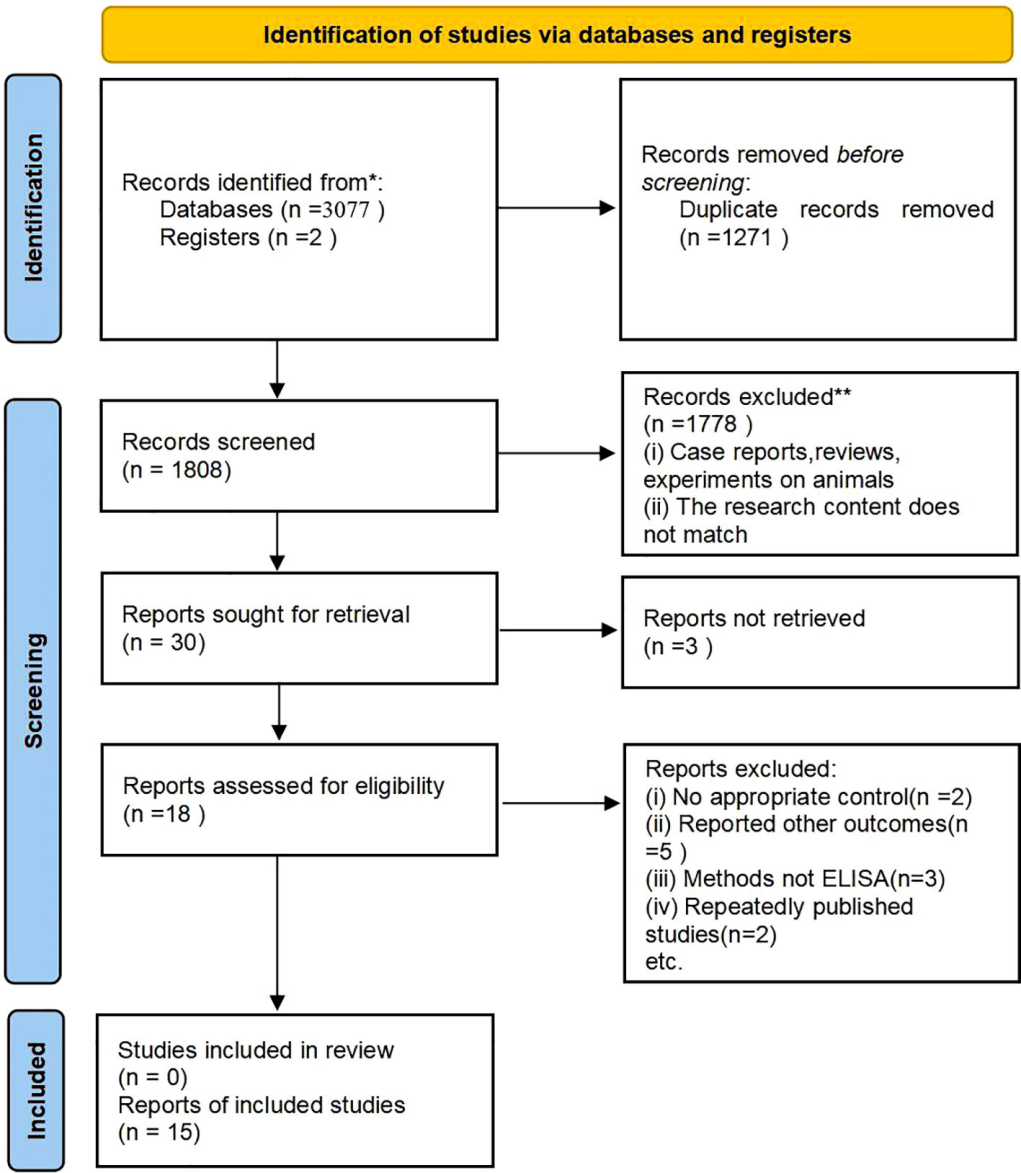
This flow chart illustrates the process of searching for literature and selecting studies. * Indicates: PubMed, Embase, Cochrane, Web of Science, Scopus, CNKI.

A total of 3,077 research papers in both Chinese and English were retrieved for the purpose of this study. Initial reading excluded duplicate studies, leaving 1808 articles. Further reading and review of titles/abstracts led to the exclusion of 1,778 articles that were found to be irrelevant to the chosen topic. Subsequently, thirty articles were selected for thorough review. A total of 15 studies were excluded for the following reasons: 3 articles did not have full text; 2 articles did not have a control group; 5 articles did not have relevant data; 3 articles had assays that were not ELISA, and 2 articles were duplicates of published studies. The exclusion process is visually depicted in **Figure 1**.

### Basic characteristics and quality of the included documents

These 15 (11, 14–27) papers collectively included 996 patients diagnosed with EMT and 582 patients without EMT. The staging system utilized for endometriosis is the revised American Society for Reproductive Medicine (rASRM) classification, which categorizes the disease into stages I-IV; stage I represents “minimal” disease, while stage IV denotes “severe” disease (28).Among the 996 EMT patients, 453 were classified as stage I-II, while the remaining 543 were categorized as stage III-IV, while 543 were categorized as stage III-IV. The fundamental characteristics of the incorporated literature are detailed in **Tables 1** and **2**.

**Table 1:** Characteristics of included studies.

Author	Year	Country	Study Type	NOS	Sample size (disease/control)	Population characteristic (disease/control)	Assay approach
Areti Augoulea1	2019	Greece	Case control	7	33/15	Age (years): 35.20±7.40/34.90±5.30 BMI (kg/m^2^): 24.40±2.08/ 25.10±2.90	ELISA
Canyu Li	2019	China	Case control	8	32/40	Age (years): 33.00±6.00/32.00±5.00	ELISA
Dongmei Man	2007	China	Case control	7	56/18	Age (years): NA	ELISA
Feng Zhu	2016	China	Case control	6	64/30	Age (years): 31.50±0.80/30.50±7.50	ELISA
Haiping Liu	2015	Italy	Case control	7	50/26	Age (years): 31.90±7.80/30.50±8.20 BMI (kg/m^2^): 23.60±3.20/24.20±4.10	ELISA
Hong Han	2017	China	Case control	9	128/50	Age (years): 43.38±8.85/42.92 ±7.68	ELISA
Jia Wang	2021	China	Case control	8	113/62	Age (years): 33.52±6.27/32.07±5.62	ELISA
Lu Li[8]	2012	China	Case control	8	45/20	Age (years): 35.40±5.80/32.60±4.60	ELISA
Suqin Liu	2003	China	Case control	8	25/16	Age (years): 34.97±6.92/37.75±4.20	ELISA
Xianning Meng	2017	China	Case control	8	100/100	Age (years): 36.80±5.10/37.10±4.90	ELISA
Xiaoyong Pan	2016	China	Case control	8	83/35	Age (years): 29.50±8.00/30.00±9.50	ELISA
Yanjun Wang	2015	China	Case control	8	40/20	Age (years): 34.65±5.63/34.80±4.98	ELISA
Yuhua Yao	2010	China	Case control	9	57/30	Age (years): 35.78±7.80/35.70±6.00	ELISA
Yuying Zhang	2013	China	Case control	8	70/20	Age (years): NA	ELISA
Zhihua Chen	2018	China	Case control	9	100/100	Age (years): 37.52±6.81/38.16±6.86	ELISA

NOS, Newcastle-Ottawa Scale; NA, not applicable; ELISA, Enzyme linked-immuno-sorbent assay; Continuous variable results are presented as means ± standard deviations (SDs).

A total of 15 literatures were included in this study, all of which were case-control studies. A total of 1578 patients were diagnosed, including 996 patients with EMT and 582 patients with non-EMT. The literature’s quality was evaluated by employing the Newcastle-Ottawa Scale scores.

**Table 2:** Characteristics of participants included in the study.

Author	Year	Endometriosis MMP-9 (Mean ± SD)	Control MMP-9 (Mean ± SD)	I-II stages MMP-9 (Mean ± SD)	I-IIstages Sample (n)	III-IV stages MMP-9 (Mean ± SD)	III-IV stages Sample (n)	Control Source
Areti Augoulea1	2019	104.21 ± 33.74	113.80 ± 32.70	113.60 ± 33.80	13	98.10 ± 33.10	20	Other diseases
Canyu Li	2019	970.13 ± 368.19	578.70 ± 246.10	600.90 ± 352.10	5	1038.50 ± 333.90	27	Uterine Fibroid
Dongmei Man	2007	297.95 ± 113.87	272.00 ± 60.34	216.05 ± 48.21	15	385.70 ± 96.92	41	Other diseases
Feng Zhu	2016	224.8 ± 88.09	142.90 ± 28.00	151.80 ± 35.20	33	302.50 ± 53.60	31	Uterine Fibroid
Haiping Liu	2015	269.34 ± 89.54	107.10 ± 20.70	211.40 ± 52.10	26	332.10 ± 78.90	24	Other diseases
Hong Han	2017	124.87 ± 64.06	42.01 ± 9.30	81.03 ± 22.24	78	193.26 ± 45.01	50	Health problem
Jia Wang	2021	15.58 ± 3.64	8.42 ± 2.15	10.19 ± 1.70	53	18.07 ± 2.26	60	Health problem
Lu Li	2012	130.7 ± 46.12	69.86 ± 18.26	89.13 ± 12.66	23	174.16 ± 20.44	22	Other diseases
Suqin Liu	2003	105.57 ± 33.56	67.10 ± 8.42	59.44 ± 10.74	8	127.28 ± 8.60	17	Other diseases
Xianning Meng	2017	233.99 ± 91.32	138.70 ± 29.10	147.80 ± 34.80	45	304.50 ± 55.20	55	Other diseases
Xiaoyong Pan	2016	290.74 ± 96.42	181.50 ± 29.50	198.30 ± 35.60	37	365.10 ± 57.00	46	Uterine Fibroid
Yanjun Wang	2015	346.94 ± 215.62	155.44 ± 83.58	202.64 ± 93.31	8	383.01 ± 223.18	32	Other diseases
Yuhua Yao	2010	37.75 ± 20.23	6.30 ± 2.10	20.60 ± 10.70	28	54.30 ± 11.40	29	Health problem
Yuying Zhang	2013	350.4 ± 104.18	207.00 ± 65.34	275.07 ± 69.21	24	389.71 ± 97.92	46	Uterine Fibroid
Zhihua Chen	2018	237.92 ± 75.5	161.71 ± 44.27	206.17 ± 60.21	57	280.00 ± 73.64	43	Health problem

NA, not applicable; Continuous variable results are presented as means ± standard deviations (SDs).

Information was collected on the patients included in the study, such as serum MMP-9 levels, and serum MMP-9 levels in patients with endometriosis who had already had different stages.

### Literature quality evaluation

The quality of the literature was assessed using Newcastle-Ottawa Scale scores. Each article scored 6 or higher, with three articles achieving a score of 9, eight articles scoring 8, three articles scoring 7, and one article scoring 6, suggesting the relatively high quality of the literature included (see **Table 1**).

### Meta-analysis

**Figure 2:**
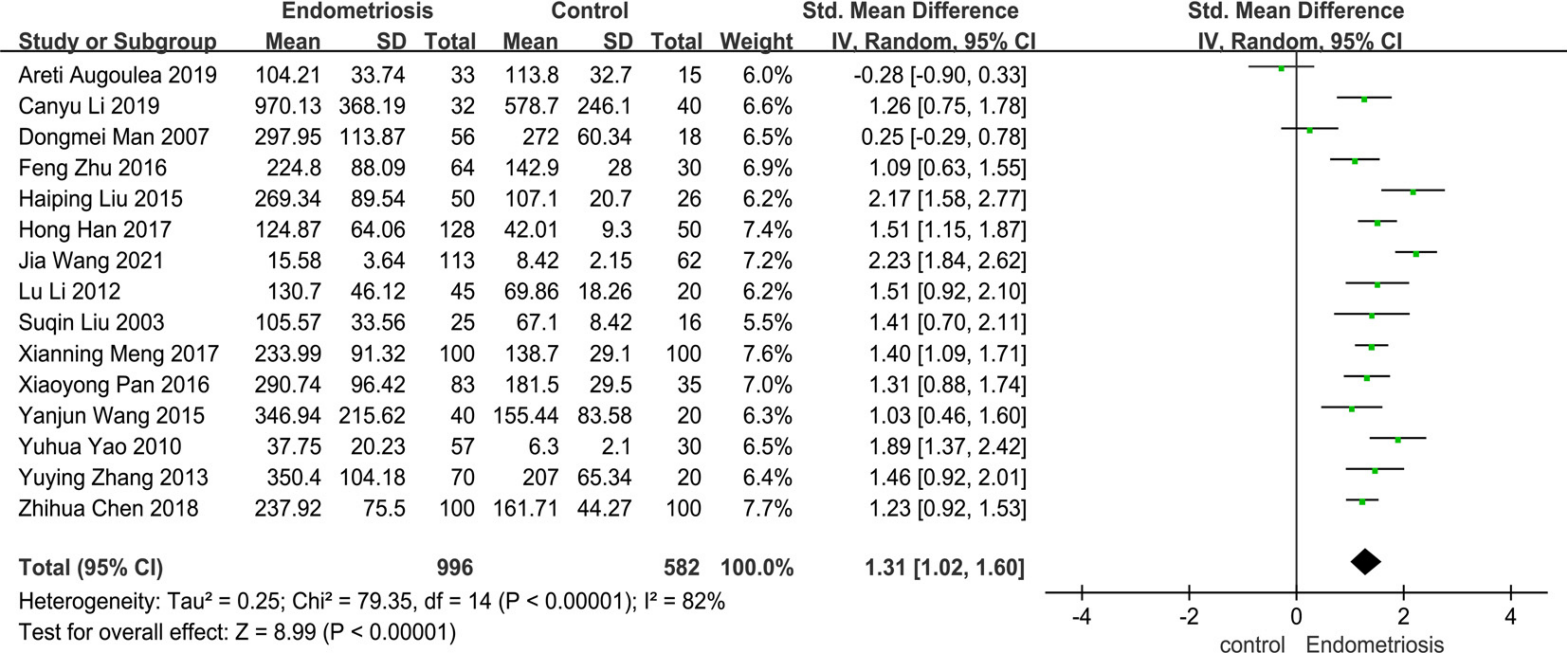
The forest plot pooled the SMD (95%) of MMP-9 level between EMT patients and controls. A total of 15 studies reported serum levels of MMP-9 in EMT and non-EMT patients, with a total of 1578 patients. There was high heterogeneity among the studies (*I^2^* = 82%, *p* < 0.00001), and the random effects model was used Analyze. The results of meta-analysis showed that serum MMP-9 level was increased after the combination. (SDM: 1.31, 95% CI: 1.02-1.60).

This study aims to assess the differences in MMP-9 levels between patients with endometriosis (EMT) and non-EMT individuals. The Forest plot presented in **Figure 2** illustrates a meta-analysis of data from 15 studies that compared serum MMP-9 concentrations in both patient groups. The findings indicate that serum MMP-9 levels were significantly elevated in endometriosis patients compared to the control group (SMD = 1.31, 95% CI = 1.02 -1.60), with a p-value < 0.00001 indicating statistical significance. However, there was considerable heterogeneity observed, as evidenced by an *I²* value exceeding 82%.

### Subgroup analyses

Subgroup analyses were performed on the 15 included articles due to the highly heterogeneous nature of the meta-analysis of the overall population.

### Analysis of subgroups based on endometriosis stage

According to the revised Endometriosis staging criteria of the American Society for Reproductive Medicine, Endometriosis is distinctly classified into stages I, II, III, and IV. Research indicates that the concentration of MMP-9 varies with the progressive severity of endometriosis (29, 30). Thus, it was imperative to stratify endometriosis patients into groups based on disease severity. We then conducted a subgroup analysis of 15 studies involving the comparison of serum MMP-9 levels in patients with different stages of endometriosis and non-endometriosis.

**Figure 3:**
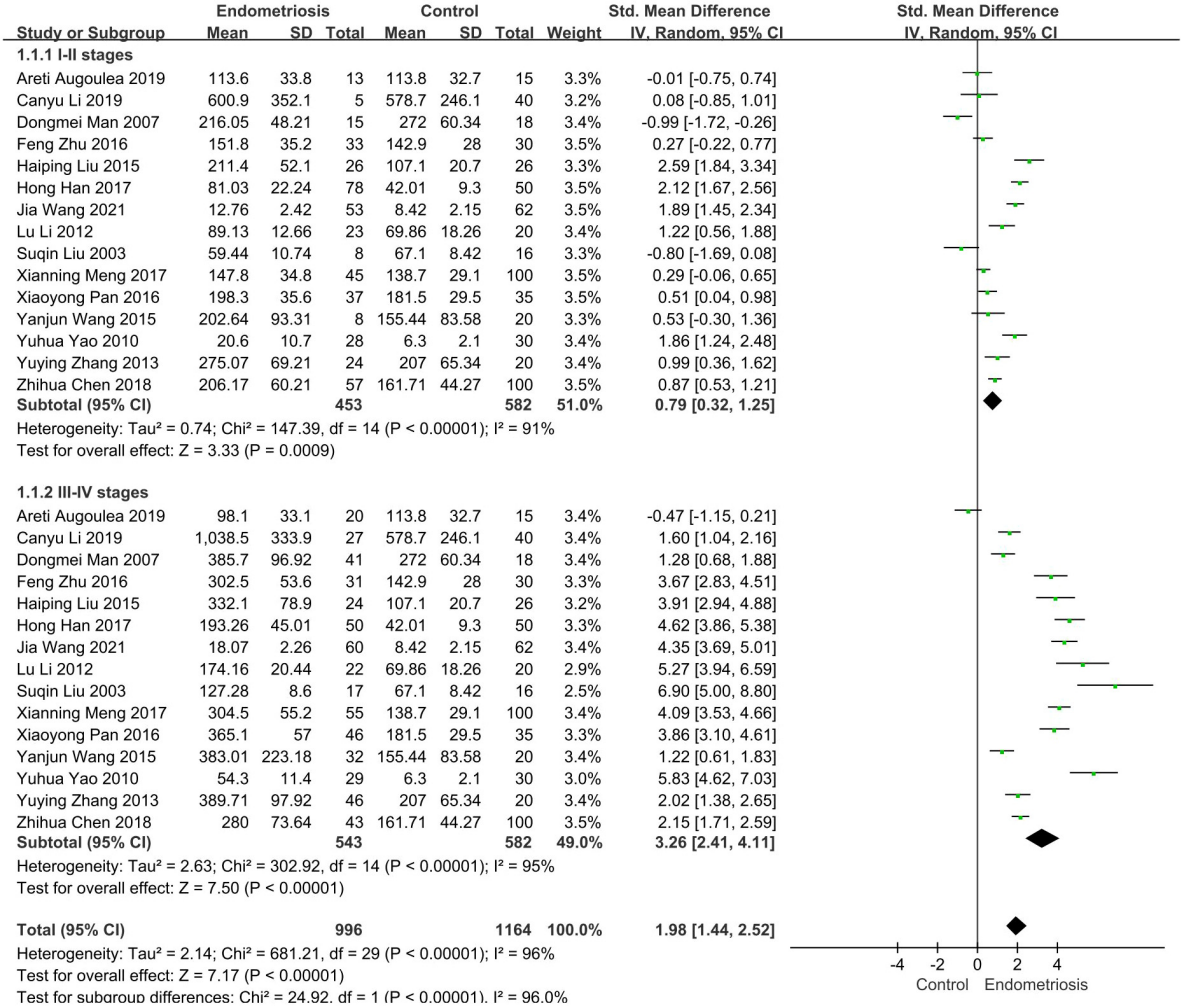
Subgroup analysis depends on different stages of endometriosis. Subgroup analysis according to different stages showed that serum MMP-9 levels were higher in 453 patients with I-II stages than in the control group (SMD= 0.79, 95% CI: 0.32-1.25, *p* < 0.00001), serum MMP-9 levels were significantly elevated in 543 patients with III-IV stages (SMD = 3.26, 95% CI: 2.41-4.11, *p* < 0.00001).

In 15 studies comparing patients with Stages I-II endometriosis with controls, we found that MMP-9 expression was elevated in patients with stage I-II endometriosis compared to controls (SMD = 0.79, 95% CI = 0.32-1.25, *I²* = 91%), a highly statistically significant difference (*p* < 0.0001). Similarly, results were observed in 15 studies involving patients with stage III-IV endometriosis: patients with Stages III-IV had significantly higher MMP-9 concentrations than controls (SMD= 3.26, 95% CI =2.41-4.11, *I²* = 95%), indicating a difference that was also highly statistically significant (*p* < 0.0001) (**Figure 3**).

### Subgroup analysis based on heterogeneity of control samples

**Figure 4:**
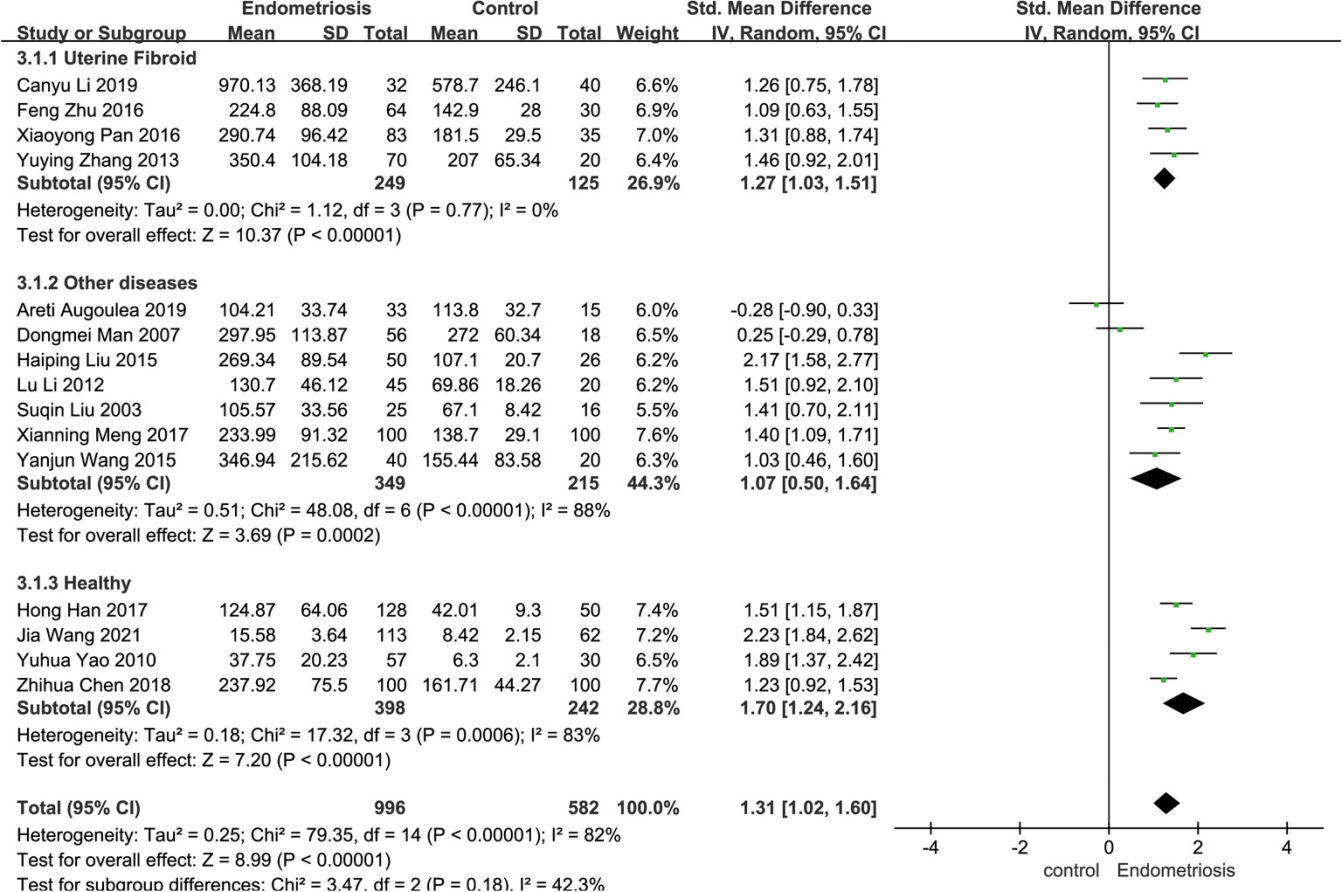
Subgroup analysis depends on different control sources. Subgroup analysis was performed to compare serum MMP-9 levels between EMT patients and different control groups. In 4 studies, patients with uterine fibroids were the control group. Seven of the studies were controlled by patients with other diseases (e.g., unexplained infertility, chronic pelvic pain, dyspareunia, patients with uterine fibroids, etc.); In four studies, the control group was healthy people.

Certain disease states, such as cardiovascular disease, cancer, and neurodegenerative diseases, have the potential to trigger significant changes in serum matrix metalloproteinases (MMPs) levels (31). In the included studies, the control samples consisted of patients with uterine fibroids, individuals with other diseases (e.g., unexplained infertility, chronic pelvic pain, dyspareunia, patients with uterine fibroids, etc.), and healthy subjects, and subgroup analyses were performed based on the heterogeneity of the control samples. The control group was categorized into uterine fibroid group, other diseases group, and healthy group. In four studies employing patients with uterine fibroids as controls, patients with endometriosis had significantly higher MMP-9 concentrations than patients with uterine fibroids (SMD = 1.27, 95% CI = 1.03-1.51, *I²* = 0%, *p* < 0.0001). Seven studies using patients with other diseases as controls also demonstrated elevated MMP-9 concentrations in endometriosis patients compared to these controls (SMD = 1.07, 95% CI = 0.50-1.64, *I²* = 88%, *p* = 0.0002). Seven studies using patients with other diseases as a control group found higher MMP-9 concentrations in patients with EMT than in these patients (SMD = 1.07, 95% CI = 0.50-1.64, *I²* = 88%, *p* = 0.0002). In four studies, the control group consisted of healthy people. Individuals with endometriosis exhibited higher MMP-9 concentrations compared to the healthy cohort (SMD = 1.70, 95% CI = 1.24-2.16, *I²* = 83%, *p* < 0.0001) (**Figure 4**).

### Sensitivity analysis

**Figure 5:**
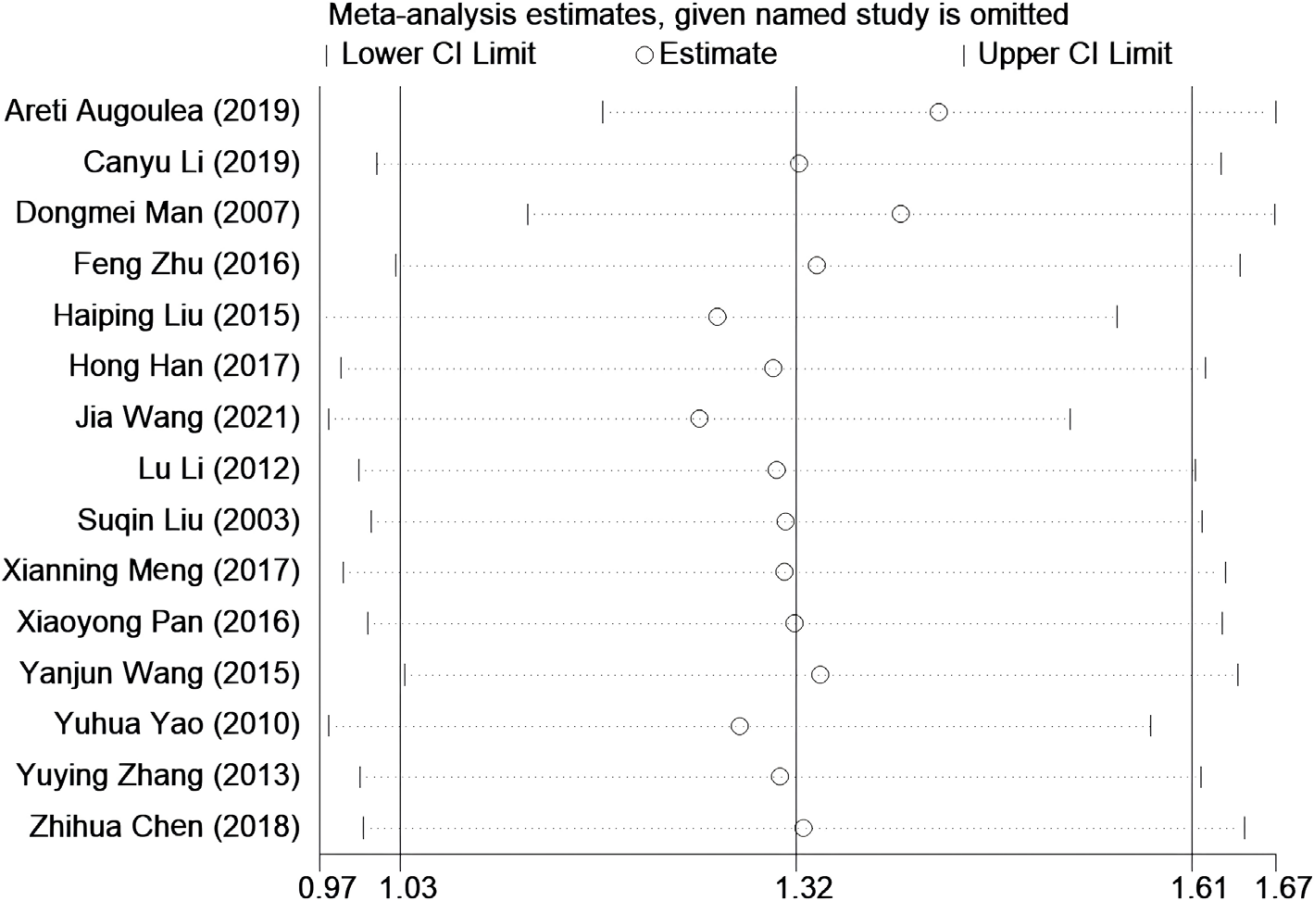
Sensitivity analysis was performed to evaluate the stability of the meta-analysis model. None of them has a greater impact on the results, that is, this result is relatively stable.

Sensitivity analysis was performed by systematically excluding 15 studies one by one. After excluding most studies, it was observed that the combined effect size of the remaining studies remained stable over the 95% confidence interval. This observation suggests that the results of the original meta-analysis are robust, as they were not significantly affected by changes in the number of studies (see **Figure 5**).

### Publication bias evaluation

**Figure 6:**
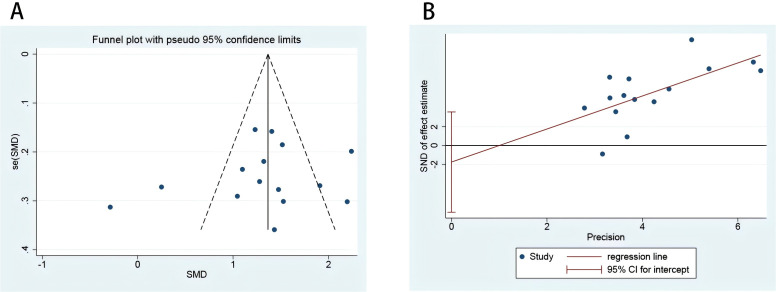
risk of bias plot of included studies. Funnel plot to evaluate the study bias of serum MMP-9 levels in EMT versus non-EMT **(A)**; Egger’s test of 15 studies included in the analysis **(B)**.

The Begg’s test and Egger’s test (**Figure 6B**) indicated no significant publication bias (*p* = 0.77, *p* = 0.50). The corresponding funnel plot (**Figure 6A**) used to assess publication bias was approximately symmetric, and further symmetry analysis yielded *p* = 0.49. Therefore, it can be concluded that there is no publication bias in the literature related to this study (**Figure 6**).

### Bioinformatic analysis

**Figure 7:**
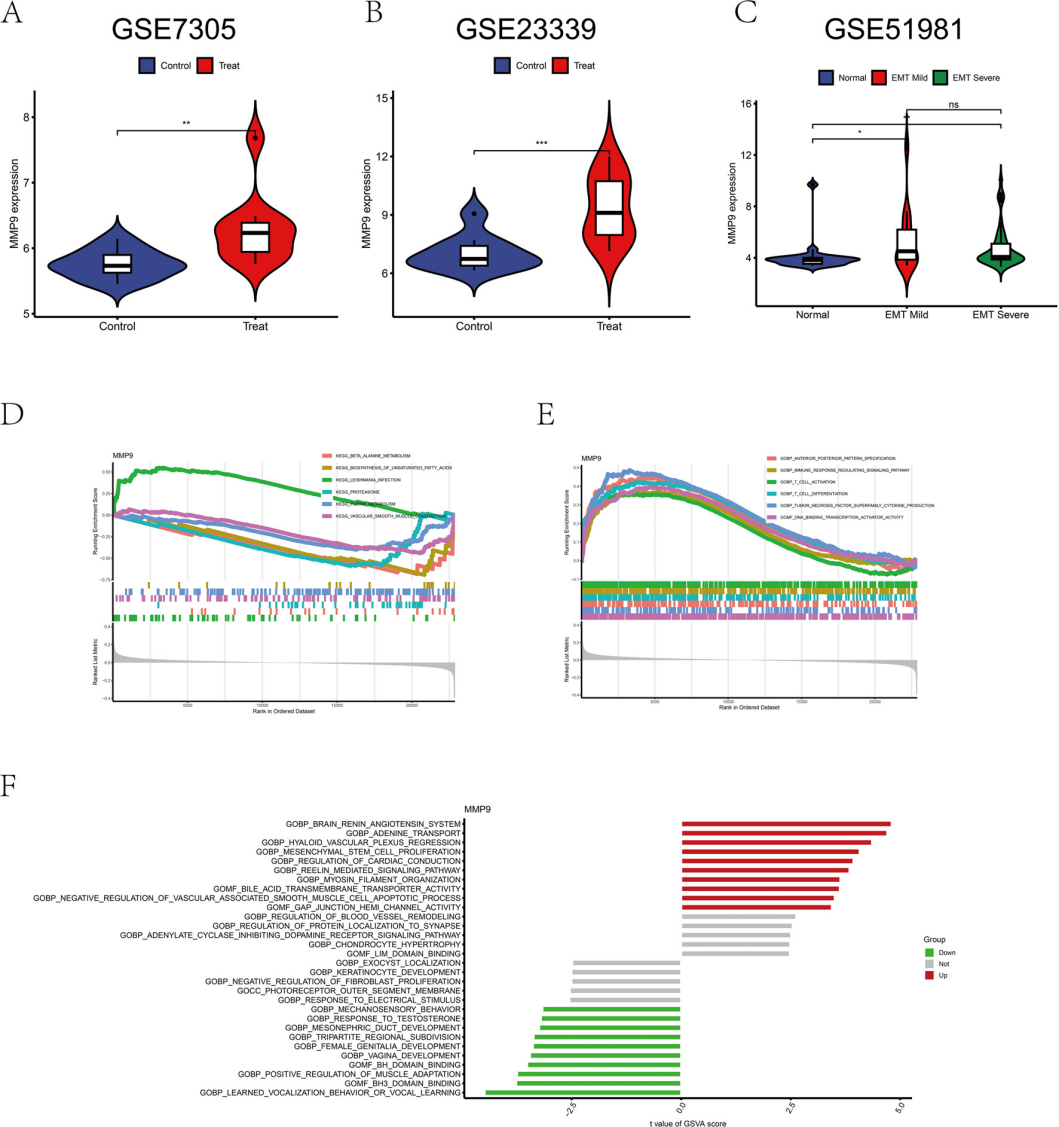
Bioinformatics analysis of MMP9 expression. **(A)** Difference analysis of MMP9 in GSE7305. **(B)** Difference analysis of MMP9 in GSE23339. **(C)** Difference analysis of MMP9 in GSE51981. **(D)** GSEA analysis of MMP9 in the pathway. **(E)** GSEA analysis of MMP9 in function. **(F)** GSVA analysis of MMP9. *p < 0.05, **p < 0.01, ***p < 0.001; ns, No significant difference.

Analysis of MMP9 gene expression in the GEO datasets GSE7305 and GSE23339 revealed a significant difference between the normal group and endometriosis samples (*p* < 0.01, *p* < 0.001), with MMP9 expression elevated in endometriosis (**Figures 7A, B**). GSE51981 data showed an increase in MMP-9 expression with increasing severity compared to controls (*p <*0.05, *p* < 0.01) (**Figure 7C**). Unfortunately, there was no difference in MMP-9 between mild and severe endometriosis in the dataset (*p* > 0.05) (**Figure 7C**). GSEA analysis indicated up-regulation of leishmania infection pathway and down-regulation of β-alanine metabolism and unsaturated fatty acid biosynthesis pathways (**Figure 7D**). Additionally, immune response regulation signaling pathways, T-cell activation, T-cell differentiation, and tumor necrosis factor superfamily cytokine production pathways were upregulated (**Figure 7E**). GSVA analysis demonstrated upregulation of MMP9 gene in the brain renin-angiotensin system, adenosine transport, MSC proliferation, and cholinesterase transmembrane transporter activity (**Figure 7F**).

## Discussion

This study showed that serum concentrations of MMP-9 were significantly higher in patients with EMT than in non-EMT patients. Meta-analysis results indicate a marked increase in MMP-9 concentration within the bodies of EMT patients compared to non-EMT patients. Specifically, the MMP-9 concentration in patients with stage III-IV EMT (SMD= 1.95, 95% CI = 1.41-2.50) is notably higher than in those with stage I-II EMT (SMD = 0.72, 95% CI = 0.30-1.15), suggesting a potential positive correlation between MMP-9 concentration and the severity of EMT. Haiping Liu et al. also believed that MMP-9 concentration was positively correlated with the severity of endometriosis and could be used as a biochemical indicator (32), which was consistent with the results of our meta-analysis. Further bioinformatics analysis revealed that the expression levels of MMP-9 in the endometrial tissue of EMT patients are significantly upregulated compared to healthy controls. However, it is noteworthy that in this study, no significant difference in MMP-9 expression levels was observed between stage I-II and stage III-IV endometriosis patients, and the underlying mechanisms warrant further investigation and clarification.

MMPs play pivotal roles in diverse biological processes including embryonic development, morphogenesis, tissue resorption, and remodeling of reproductive tissues (33). Moreover, MMPs contribute significantly to pathological processes including inflammation, arthritis, cardiovascular diseases, pulmonary ailments, and cancer. Therefore, we performed a subgroup analysis that showed higher concentrations of MMP-9 in patients with endometriosis (EMT) compared to patients with uterine fibroids, patients with other diseases, and healthy individuals. This discovery suggests that MMP-9 could serve as a potential biomarker for distinguishing endometriosis from other conditions. Areti Augoulea and her team (14) collected serum samples from 31 patients diagnosed with EMT and compared them with serum samples from 15 patients admitted to the same hospital for non-EMT-related conditions. The results showed that the serum MMP-9 content in EMT patients was significantly higher than that in the control group. Similarly, the study by Yanjun Wang et al. (24) used serological testing to compare serum samples from 40 EMT patients with those from infertile patients diagnosed with hydrosalpinx at the same time. The results consistently showed that serum MMP-9 levels were higher in EMT patients than in infertile patients with hydrosalpinx, further supporting the potential role of MMP-9 in the pathological process of EMT. Therefore, the results of the meta-analysis presented here may provide an important reference for evaluating the diagnostic utility of MMP-9 in endometriosis in the future.

By GSEA pathway enrichment analysis, we found that MMP-9 was significantly enriched in the unsaturated fatty acid pathway and tended to be downregulated in endometriosis. It has been shown that prostaglandins are important metabolites biosynthesized from polyunsaturated fatty acids (PUFAs), of which arachidonic acid is the main source (34–36). Prostaglandin E2 (PGE2) is a potent inducer of Steroidogenic Acute Regulatory (StAR) protein and aromatase within the stromal cells of endometriosis, thereby facilitating the aberrant production of estrogens to sustain the survival and proliferation of ectopic endometriotic tissues (37). Furthermore, Parazzini et al. have proposed a multifactorial etiology implicating genetic traits, inflammatory responses, hormonal fluctuations, menstrual patterns, organochlorine exposure, prostaglandin metabolism, and immunological factors in the establishment and progression of endometriosis (38). Studies conducted on human dermal fibroblasts reveal that PGE2 inhibits Transforming Growth Factor-beta 1 (TGF-β1)-induced collagen synthesis by modulating the balance between MMPs and Tissue Inhibitors of Metalloproteinases (TIMPs) (38). Conversely, in the presence of TGF-β1, PGE2 upregulates the expression of MMP-2/MMP-9 while downregulating TIMP-1 expression. This aligns with our observation of elevated MMP-9 expression in endometriosis patients, consistent with our findings. It can be speculated that MMP-9 may become a diagnostic marker in serum samples for endometriosis.

GSEA enrichment analysis showed that high expression of MMP-9 would activate the tumor necrosis factor family pathway. It has been demonstrated that TNF augments the proliferation of both normal and ectopic endometriotic cells (39). Aberrant TNF levels have been linked to various reproductive disorders (40–42). Studies have demonstrated that macrophage recruitment and its polarized phenotype in endometriotic lesions contribute to both the development and maintenance of endometriosis. TNF-α stimulates macrophage production of MMP-9 (43). The levels of MMP9 were significantly different in the blood and tissues of both normal and endometriotic groups. We hypothesize that TNF-stimulated macrophage production of MMP9 represents one potential mechanism underlying the generation and maintenance of endometriosis.

There are several advantages to testing serum MMP-9 levels. First, MMP-9 concentrations can be used as an initial assessment of a patient’s condition if the patient declines invasive testing or surgery. Secondly, ultrasound imaging has certain limitations in the observation of deep lesions and complex anatomical structures, especially for deep pelvic lesions, such as rectal and bladder involvement, the resolution of ultrasound imaging is low and it is difficult to clearly display (44, 45). To overcome these shortcomings, several physiologic markers have been proposed in clinical practice to help determine prognosis, including CA125 and CA199 markers (46–48). Currently, clinical serum markers (like MMP-9) do not replace existing physiological measures to predict patient prognosis; however, these tests and methods have their limitations. Combined testing based on physiologic indicators, imaging, and serum markers more accurately predicts prognosis in patients with EMT (49, 50).

Although stratified analyses have been carried out to every extent possible, the present meta-analysis still has certain shortcomings. Firstly, the absence of randomized controlled trials or prospective cohort studies with large sample sizes might diminish the credibility of the study’s findings. Secondly, the different pathophysiological processes of endometriosis, such as proliferative and secretory phases, may have an important impact on the heterogeneity of the disease. Unfortunately, due to a lack of detailed reporting or missing information in the literature, we were unable to conduct a comprehensive subgroup analysis to quantify the impact of these key factors on disease heterogeneity, and this study was limited to English and Chinese language publications. As a result, other studies published in journals in other languages may be ignored, leading to a bias in the conclusions. This situation undoubtedly compounds the challenge of comprehensively understanding and treating endometriosis. Therefore, we sincerely hope that forthcoming research will accurately measure MMP-9 concentrations in the serum of endometriosis patients during both proliferative and secretory phases, categorizing them accordingly, to delve deeper into their pathophysiological mechanisms. This publication may offer guidance for future studies, particularly regarding the dose-response relationship between elevated MMP-9 concentrations and EMT progression. Future studies may use the MMP-9 concentration threshold as a direction for diagnosing EMT to prompt physicians to intervene early in progressive EMT.

## Conclusions

The results of the meta-analysis suggest that elevated serum levels of MMP-9 in patients with endometriosis may serve as a potential diagnostic marker, but more high-quality studies are needed to confirm these effects.

This study may provide important insights for clinical diagnosis and treatment of endometriosis.

## Data Availability

The datasets presented in this study can be found in online repositories. The names of the repository/repositories and accession number(s) can be found in the article/**Supplementary Material**.
