# Supplementary material for: MMP-9 as a clinical marker for endometriosis: a meta-analysis and bioinformatics analysis
Source: Front Endocrinol (Lausanne). 2024 Oct 31;15:1475531. doi: 10.3389/fendo.2024.1475531 (PMC11560754; doi:10.3389/fendo.2024.1475531)
Supplement: Supplementary file 1 [file DataSheet1.docx]

**Appendix A**

1. **Pubmed Search Strategy**

**Searched March 6, 2024.**

| #1"Endometriosis"[MeSH Terms](26223) |
| --- |
| #2"Endometrioses"[Title/Abstract] OR "Endometrioma"[Title/Abstract] OR "Endometriomas"[Title/Abstract](3210) |
| #3"Endometriosis"[MeSH Terms] OR "Endometrioses"[Title/Abstract] OR "Endometrioma"[Title/Abstract] OR "Endometriomas"[Title/Abstract](26995) |
| #4"Matrix Metalloproteinases"[MeSH Terms](53579) |
| #5"metalloproteinases matrix"[Title/Abstract] OR "MMPs"[Title/Abstract] OR "matrix metalloproteinase"[Title/Abstract] OR "metalloproteinase matrix"[Title/Abstract] OR "MMP"[Title/Abstract](81938) |
| #6"Matrix Metalloproteinases"[MeSH Terms] OR "metalloproteinases matrix"[Title/Abstract] OR "MMPs"[Title/Abstract] OR "matrix metalloproteinase"[Title/Abstract] OR "metalloproteinase matrix"[Title/Abstract] OR "MMP"[Title/Abstract](91600) |
| #7("Endometriosis"[MeSH Terms] OR ("Endometrioses"[Title/Abstract] OR "Endometrioma"[Title/Abstract] OR "Endometriomas"[Title/Abstract])) AND ("Matrix Metalloproteinases"[MeSH Terms] OR ("metalloproteinases matrix"[Title/Abstract] OR "MMPs"[Title/Abstract] OR "matrix metalloproteinase"[Title/Abstract] OR "metalloproteinase matrix"[Title/Abstract] OR "MMP"[Title/Abstract]))(380) |

1. **Embase Search Strategy**

**Searched March 6, 2024.**

#1. 'endometriosis'/exp OR endometriosis (56503)

#2.'endometrioses':ab,ti OR 'endometrioma':ab,ti OR 'endometriomas':ab,ti（5160）

#3. #1 OR #2 （57692）

#4. matrix AND metalloproteinases （35253）

#5. 'metalloproteinases, matrix':ab,ti OR 'mmps':ab,ti OR 'matrix metalloproteinase':ab,ti

OR 'metalloproteinase, matrix':ab,ti OR 'mmp':ab,ti （107456）

#6. #4 OR #5（115727）

#7. #3 AND #6 （589）

1. **Cochrane Search Strategy**

**Searched March 6, 2024.**

#1 Endometriosis (3305)

#2 (Endometrioses):ab,ti,kw OR (Endometrioma):ab,ti,kw OR (Endometriomas):ab,ti,kw (419)

#3 #1 OR #2 (3417)

#4 Matrix Metalloproteinases (726)

#5 (Metalloproteinases, Matrix):ab,ti,kw OR (MMPs):ab,ti,kw OR (Matrix Metalloproteinase):ab,ti,kw OR (Metalloproteinase, Matrix):ab,ti,kw OR (MMP):ab,ti,kw (3179)

#6 #4 OR #5 (3216)

#7 #3 AND #6 (11)

1. **Web of Science Search Strategy**

**Searched March 6, 2024.**

| #1 TS=(Endometriosis OR Endometrioses OR Endometrioma OR Endometriomas) (32362) |
| --- |
| #2 TS=(Matrix Metalloproteinases OR Metalloproteinases, Matrix OR MMPs OR Matrix Metalloproteinase OR Metalloproteinase, Matrix OR MMP)(117205) |
| #1 AND #2 (647)  **CNKI Search Strategy**  **Searched March 6, 2024.**  #1.(Topic: Endometriosis) AND (Topic: MMP) (765) |
